# Melatonin Supplement Plus Exercise Effectively Counteracts the Challenges of Isoproterenol-Induced Cardiac Injury in Rats

**DOI:** 10.3390/biomedicines11020428

**Published:** 2023-02-01

**Authors:** Md. Mahbubur Rahman, Dong Kwon Yang

**Affiliations:** 1 Department of Veterinary Pharmacology and Toxicology, College of Veterinary Medicine, Jeonbuk National University, Iksan 54596, Republic of Korea; 2 Department of Physiology, College of Medicine, Gachon University, Incheon 21999, Republic of Korea

**Keywords:** antioxidant, cardiac injury, exercise, isoproterenol, melatonin, oxidative stress

## Abstract

To explore the combined effects of exercise and melatonin supplement against the challenges of isoproterenol-induced cardiac oxidative stress and injury in rats., the expression of peroxisome proliferator-activated receptor gamma co-activator-1α (PGC-1α), mitochondrial biogenesis, and adenosine triphosphate (ATP) was up-regulated in cardiac muscle in normal rats and in a melatonin and exercise regimented group. Cardiac injury was induced by two subcutaneous injections of isoproterenol in the rats. The combination of exercise and melatonin supplement successfully counteracted the isoproterenol-induced cardiac injury, which is reflected by the improved hemodynamic parameters, reduction in oxidative stress markers, and cardiac injury serum markers (cardiac troponin-I and creatine kinase-MB). The cardiac tissue level of ATP, expression of PGC-1α and mitochondrial biogenesis-related genes, mitochondrial membrane potential, and the activities of typical antioxidants (glutathione, superoxide dismutase) were preserved, whereas the levels of reactive oxygen species, lipid peroxidation, and inflammatory cytokines were suppressed in the melatonin and exercise regimented (MEI) group compared to the group treated with isoproterenol alone. Furthermore, the expression of endoplasmic reticular stress- and apoptosis-related proteins (Bax, Bcl2, and caspase-3) was also effectively suppressed in the MEI group. Therefore, the present study suggests that melatonin supplement in combination with exercise prevents cardiac injury, possibly through the preservation of mitochondrial function and inhibition of oxidative stress in rats.

## 1. Introduction

The leading causes of morbidity and mortality have been reported to be non-communicable diseases such as aging, obesity, and diabetes, especially the cardiovascular diseases due to dietary behavior (higher dietary glycemic and trans-fat intake, tobacco and alcohol consumption), and the modern socioeconomic and technology-based lifestyle that has reduced laborious activities or increased physical inactivity [1,2]. Cardiovascular disease is a global public health concern that causes 30% of global mortality and 10% of the global disease burden [3]. Myocardial infarction is a common feature of ischemic cardiovascular disease. Public awareness of myocardial infarction has been raised because it still remains the leading cause of sudden death, even though clinical care has improved. Isoproterenol, a synthetic catecholamine and β-adrenergic agonist, has been reported to induce cardiac dysfunction resulting from myocardial ischemia, hypoxia, and oxidative stress, and further cause necrosis, which closely resembles local myocardial infarction/ischemia-like pathological changes and heart failure in human myocardial infarction [4,5,6].

Cardiac oxidative balance is strictly maintained by the affluence of oxidant and antioxidant systems that regulate the production and removal of reactive oxygen species (ROS). Overproduction of ROS impedes oxidative defense and plays a critical role in cardiac failure and arrest by triggering pathological process such as necrosis, ER stress, mitochondrial dysfunction, apoptosis, ionic imbalance, and ATP reduction [7], and also down-regulates peroxisome proliferator-activated receptor-γ coactivator (PGC)-1α, which is a regulator of cardiac oxidative phosphorylation and ROS detoxification [8]. Physical exercise is believed to have many beneficial effects on the incidence of cardiovascular diseases, obesity, and diabetes; conversely, a sedentary lifestyle has been recognized as a risk factor of many diseases [2,9]. Although acute exercise induces a transient oxidative stress, regular controlled exercise results in an increase in oxidative capacity and antioxidant activities [2,10,11]. Therefore, it is frequently recommended as an important tool to promote optimal health and life expectancy. Melatonin, which is primarily produced by the pineal gland, is correlated with a variety of biological processes including cardiovascular, immune, and neuroendocrine functions, and circadian rhythms, as well as thermoregulation, for its direct or indirect powerful anti-oxidative, anti-inflammatory effects, and enhancing capacity of mitochondrial activity [2,5,12,13]. In addition, melatonin [14] and exercise [15] up-regulate cardiovascular anti-oxidative defense systems and reduce oxidative stress-inducible markers.

Despite focusing on the invention of drugs for the treatment of cardiac diseases, exercise and lifestyle modifications should be emphasized to reduce their incidence because it is an important underlying factor of diseases in modern life. Therefore, the purpose of this study was to investigate the counteracting effects of melatonin administration and exercise regimentation against the challenges of isoproterenol-induced cardiac injury, oxidative stress, and myocardial dysfunction in rats.

## 2. Materials and Methods

### 2.1. The Animals and Experimental Model

A total of eighty ten-week-old male Sprague Dawley rats (252 ± 2 g, Orient Bio, Gapyeong, Gyeonggi-do, Republic of Korea) were used for this study. The rats were reared in an environment-controlled housing system (temperature, 23 ± 2 °C; humidity, 50 ± 5%; and 12 to 12 h light–dark cycle). Food and water were available ad libitum before the start of the experiment. After acclimatization for 7 days, the rats were equally divided into eight groups (n = 10): normal control (NC); melatonin control (MC); exercise control (EC); melatonin and exercise control (MEC); isoproterenol control (IC); exercise and isoproterenol (EI); melatonin and isoproterenol (MI); and melatonin, exercise, and isoproterenol (MEI) groups.

Freshly prepared melatonin (Sigma Chemical Co., St Louis, MO, USA) dissolved in 0.1% ethanol was administered daily (10 mg/kg) by oral gavage in the melatonin-treated groups [2,16,17]. The NC, IC, and EC groups were orally administered 0.1% ethanol solution of the same volume twice a day. Exercise was regimented (EC, EI, and MEI groups) in a specially designed swimming pool as described previously [2]. The temperature of the water was maintained at 36 ± 1 °C via a thermostatically controlled heater located at the base of the chamber. Water bubbles were sprangly produced by tubes connected to an air pump system to prevent floating during swimming. The rats were forced to swim 40 min/day 5 days/week for 3 weeks. After 3 weeks, the rats in the NC, EC, MC, and MEC groups were sacrificed to evaluate the effects of regular exercise and melatonin in normal rats, but the rats in the IC, EI, MI, and MEI groups were administered isoproterenol (85 mg/kg) by subcutaneous injection twice at 24 h intervals on the 22nd and 23rd day to induce cardiac injury [18], and on the 24th day, rats of all groups were sacrificed, and samples were harvested.

Before sacrificing, all rats were anaesthetized with a Zoletil 50 (Teletamine HCl, 125 mg and Zolezepum 125 mg) (Virbac Laboratories, Carros, France) and 2% Rompun (Xylazine Hcl) (Bayer, Leverkusen, Germany) combination (3:1) by intraperitoneal injection (1 mL/kg) [2]. A cannula (outer and inner 0.96 mm and 0.58 mm, respectively) (fisher Scientific) was filled with heparinized saline and inserted in the right carotid artery and connected to a Biopack computerized transducer system (Biopack, Goleta, CA, USA) for the measurement of hemodynamic parameters such as heart rate (HR), systolic blood pressure (SBP), diastolic blood pressure (DBP), and mean arterial blood pressure (MABP) using acknowledge 3.5 software (East Palo Alto, CA, USA) [2]. Left ventricular end-diastolic pressure (LVEDP), left ventricular end-systolic pressure (LVESP), and +/− left ventricular developed pressure (+/− LVdpdt) were measured by inserting the cannula in the left ventricles. All the procedures were approved by the Institutional Animal Care and Use Committee of Jeonbuk National University (JBNU 2020-084).

### 2.2. Measurement of Oxidative Stress-Associated Markers in Cardiac Tissue

To measure reactive oxygen species (ROS), fresh left ventricles of cardiac tissues were homogenized on ice with 1 mM ethylenediaminetetraacetic acid (EDTA)-50 mM sodium phosphate buffer (pH 7.4). Then, samples were incubated at 37 °C with 5 μM 2′-7′-dichlorofluorescin-diacetate (DCFH-DA) (Sigma, St Louis, MO, USA) in the dark for 30 min. Fluorescence was measured every 15 min for 1 h with excitation and emission by using 488 nm and 525 nm wavelengths, respectively, with a SpectraMax Gemini EM microplate reader (Molecular Devices, Sunnyvale, CA, USA). Relative fluorescence units (RFU) were used to express the value. Cardiac tissue concentrations of malondialdehyde (MDA) were detected with an OXI-TEK TBARS kit (Enzo Life Sciences, Farmingdale, NY, USA). Reaction products were quantified by measuring the absorbance at 532 nm according to the manufacturer’s suggested protocol. The tissue levels of superoxide dismutase (SOD) were quantified using a SOD activity kit (Sigma) by measuring the absorbance at 450 nm. The levels of glutathione (GSH) were measured at 405 nm as the absorbance of the reaction product using a glutathione detection kit (Sigma).

### 2.3. Measurement of Biochemical Parameters in Serum and Cardiac Tissues

Blood was collected from the caudal vena cava immediately following sacrifice, and serum was further separated by centrifugation at 3000 rpm/min for 10 min and stored at −20 °C until analysis. Serum levels of creatine kinase (CK), total cholesterol (TC), high-density lipoprotein (HDL), low-density lipoprotein (LDL), and triglyceride (TG) were determined with a Hitachi 7180 (Hitachi, Tokyo, Japan). Athererogenic index (AI) was calculated using TC/HDL [2]. The assessment of serum cardiac troponin-I (cTn-I) enzyme-linked immunosorbent assay (ELISA) kit and rat creatine kinase MB (CK-MB) isoenzyme ELISA kit were purchased from CUSABIO company (Shanghai, China). ATP levels in fresh left ventricles of cardiac tissue homogenates were determined calorimetrically by using an ATP assay kit (Abcam, Brandford, CT, USA) according to the manufacturer’s instructions.

For the ELISA analysis, the homogenate was centrifuged for 15 min at 9000× *g* at 4 °C and the supernatant fraction was then subjected to ELISA analysis for the measurement of tumor necrosis factor (TNF)-α protein with an ELISA kit (ALPCO Diagnostics, New Hampshire, NH, USA) following the manufacturer’s instructions.

### 2.4. Measurement of Gene Expression in Cardiac Tissues

The alteration of gene expression levels in cardiac tissues, including peroxisome proliferator-activated receptor gamma co-activator 1a (PGC-1α) [2], mitochondrial transcription factor (mtTFA) [2], nuclear respiratory factor 1 (NRF1) [2], C/EBP homologous protein (CHOP) [19], activating transcription factor 4 (ATF4) [19], Bcl-2 [20], and Bax [20], was measured using a quantitative real-time polymerase chain reaction (qRT-PCR). Trizol reagent (Invitrogen, Cergy Pontoise, France) was used according to the manufacturer’s protocol for the extraction of total RNA from the frozen left ventricles of cardiac tissues. cDNA reverse transcription kits (Applied Bio-systems, CA, USA) were used to synthesize cDNA from isolated total RNA. qRT-PCR was performed using cDNA with the SYBR Primix Ex Taq (TaKaRa, Kyoto, Japan) on a CFX96TM real-time PCR detection system (Bio-Rad Laboratories, Hercules, CA, USA). The reaction volume was 20 mL and the final working concentrations of all primers were 200 nmol/L. The obtained qPCR data were analyzed through the ΔΔCT method using a housekeeping gene glyceraldehyde-3-phosphate dehydrogenase (GAPDH) by using Bio-Rad CFX Manager Ver. 2.1 software (Bio-Rad Laboratories, Hercules, CA, USA). Product specificity was confirmed by melting or dissociation curve analysis. Primer sequences are presented in Table 1.

### 2.5. Cardiac Mitochondrial Function Measurement

Cardiac mitochondria were isolated from the left ventricle of four randomly selected rats from each group as previously described [21]. Half of the isolated mitochondria were incubated for 30 min at 37 °C in a PBS containing 2 μmol of 2′-7′-dichlorofluorescin-diacetate dye, and fluorescent excitation and emission were detected using 480 and 530 nm wavelengths, respectively. The values were calibrated according to protein concentration of the samples and demonstrated as mitochondrial ROS generation. The ratio of JC-1 monomers and JC-1 polymers represented the mitochondrial membrane potential of the cardiac mitochondria. The remaining portion of isolated mitochondria were incubated in 2 mL of PBS containing 2 μL of JC-1 dye (Sigma-Aldrich, St Louis, MO, USA) for 30 min at 37 °C in a dark environment. The wavelengths of excitation and emission used to measure the monomeric form of JC-1 were 514 nm and 529 nm, respectively, and 585 nm and 590 nm, respectively, for polymers of JC-1 detection with a SpectraMax Gemini EM microplate reader (Molecular Devices, Sunnyvale, CA, USA). Then, the ratio was calculated to demonstrate mitochondrial membrane potential (MMP).

### 2.6. Histology, Immunohistochemistry, and TUNEL Assay

For the histological analysis, the hearts were dissected from the rats in the NC, IC, EI, MI, and MEI groups (n = 4 in each group) at the end of experiment period. The tissues were washed in normal saline and fixed in 10% neutral buffered formalin solution. After fixation, the samples were cleaned and embedded in paraffin. The tissue sections thickened with 4 µm were mounted on slides, and further subjected to hematoxylin—eosin (H&E) staining for histopathological analysis, and examined on a light microscope from three separate visual fields at ×200 magnification with the same location of the left ventricle for all animals. The injury condition was scored by two individual pathologists. The 0–4-point scoring system was: 0, no lesions; 1, cells arranged roughly in order with mild necrosis and edema; 2, slightly disordered cell arrangement with moderate local necrosis and swelling; 3, disordered cell arrangement with severe necrosis and inflammatory infiltration; and 4, extremely disordered cell arrangement with very severe diffuse necrosis and inflammatory infiltration [22].

For the immunohistochemistry analysis, the paraffin sections were deparaffinized with xylene and dehydrated in a graded series of ethanol, and finally subjected to antigen retrieval in 0.01M citrate buffer at 115–120 °C for 15 min. After they were allowed to cool down to room temperature, they were washed in TBST then incubated in 0.03% H_2_O_2_ in 0.01 M PBS for 15 min at room temperature, and, in turn, incubated with 4% BSA + dextran for 30 min at 37 °C. Sections were next incubated for 1 h at 37 °C with primary antibody against caspase 3 protein (1:400, Abcam). Negative controls were incubated with only PBS. They were then rinsed with PBS and incubated with Envision anti-Rabbit secondary antibody (Dako, Santa Clara, CA, USA) for 30 min. The reaction was visualized with a diaminobenzidine (DAB). For quantification, the integral optical density (IOD) of Caspase-3 staining was calculated with computerized Image-Pro Plus 7.0 software (Media Cybernetics Inc., Silver Spring, MD, USA) [23].

TUNEL assay of cardiac tissues was performed with an ApopTag^®^ Peroxidase In Situ Apoptosis Detection Kit (Millipore, Billerica, MA, USA). After incubating with proteinase K for 10 min, they were further incubated with 3% H_2_O_2_ in methanol solution for 15 min to inactivate the endogenous peroxidase activity. TdT was added and incubated overnight at room temperature. After incubating with DAB and hydrogen peroxide, followed by counterstaining with methyl green, the apoptotic cells were shown by dark brown colors. Percentages of positive TUNEL-stained cells within cardiac areas were estimated.

### 2.7. Statistical Analysis

Prism 5.03 software (GraphPad Software Inc., SanDiego, CA, USA) was used for the statistical analysis of the data. Results are expressed as mean ± standard error of the mean (SEM). All data were analyzed using one-way analysis of variance (ANOVA) followed by the post hoc Bonferroni test. The level of significance was set at *p* < 0.05.

## 3. Results

### 3.1. Effect of Exercise and Melatonin on Body Weight, Heart Weight, and Cardiac Function

**Figure 1:**
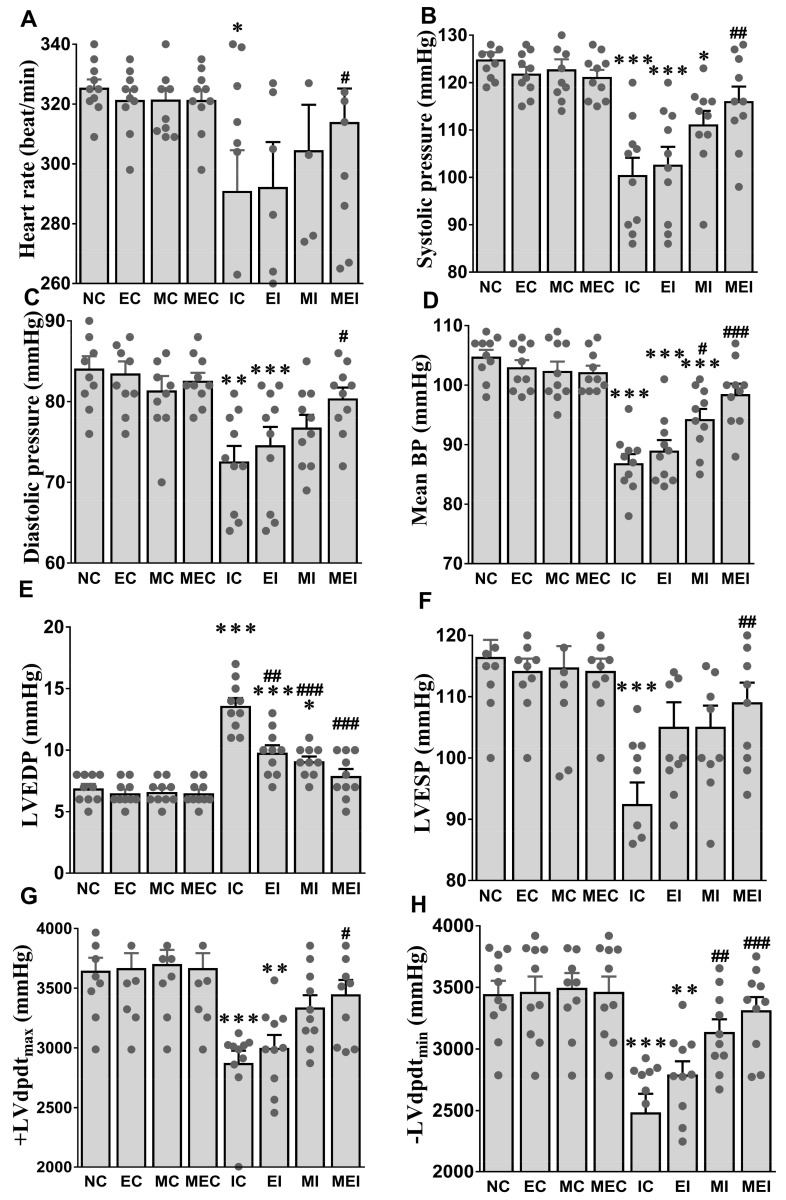
Effect of melatonin and exercise on cardiac contractile function in isoproterenol-challenged rats. (**A**) Heart rate; (**B**) Systolic pressure; (**C**) Diastolic pressure; (**D**) Mean blood pressure; (**E**) LVEDP, left ventricular end-diastolic pressure; (**F**) LVESP, left ventricular end-systolic pressure; (**G**) LVdpdt_max_, maximum left ventricular developed pressure; (**H**) LVdpdt_min_, minimum left ventricular developed pressure. The data are reported as the mean ± SEM (*n* = 10). * *p* < 0.05, ** *p* < 0.01, and *** *p* < 0.001 versus the NC group; # *p* < 0.05, ## *p* < 0.01, and ### *p* < 0.001 versus the IC group calculated through one-way ANOVA followed by the post hoc Bonferroni test. NC, normal control; EC, exercise control group; MC, melatonin control group; MEC, melatonin plus exercise control group; IC, isoproterenol control group; EI, exercise plus isoproterenol-challenged group; MI, melatonin plus isoproterenol-challenged group; MEI, melatonin and exercise plus isoproterenol-challenged group.

The exercise regimentation significantly lowered the final body weight in the EC, MEC, EI, and MEI groups compared to the sedentary NC groups. The percentage of heart and left ventricle weight to body weight (17% and 24% increases, respectively) and left ventricle weight to heart weight (6% increase) were significantly increased in the isoproterenol-challenged IC group compared to the NC group (Table 2). These alterations were markedly corrected by the combined action of melatonin and exercise in the MEI group. We did not find any significant alteration of cardiac contractile functions in the non-isoproterenol-treated NC, EC, MC, and MEC groups after 3 weeks. However, these contractile parameters, including HR, SAP, DAP, MAP, LVESP, LVEDP, +dpdt, and −dpdt, were markedly decreased in the IC group. Interestingly, the combination of melatonin and exercise significantly suppressed these alterations, which is evidenced by the improved contractile function in the MEI group compared to the IC group (Figure 1).

### 3.2. Effect of Exercise and Melatonin on Biochemical Parameters in Serum and Cardiac Tissue

Cardiac injury-related serum markers, including cTn-I, CK-MB, CK, and CK index, were significantly elevated in the IC group compared with those in the NC group. Furthermore, the CK-index level in the EI, MI, and MEI groups was lowered by 18, 15, and 21%, respectively, and the cTn-I level was reduced by 26, 37, and 57%, respectively, compared with those in the IC group (Table 3). Notably, significant differences were not observed in rats treated with melatonin or exercise alone or both melatonin supplement and exercise compared to normal control rats. Although the serum lipid profiles, including TC, HDL, LDL, TG, and AI, were ameliorated in these groups, mostly in the MEC group, when compared with the NC group, we did not observe any marked alteration of lipid profiles after 24 h in the IC group (Table 3).

### 3.3. Effect of Exercise and Melatonin on Oxidative Stress-Associated Markers in Cardiac Tissue

**Figure 2:**
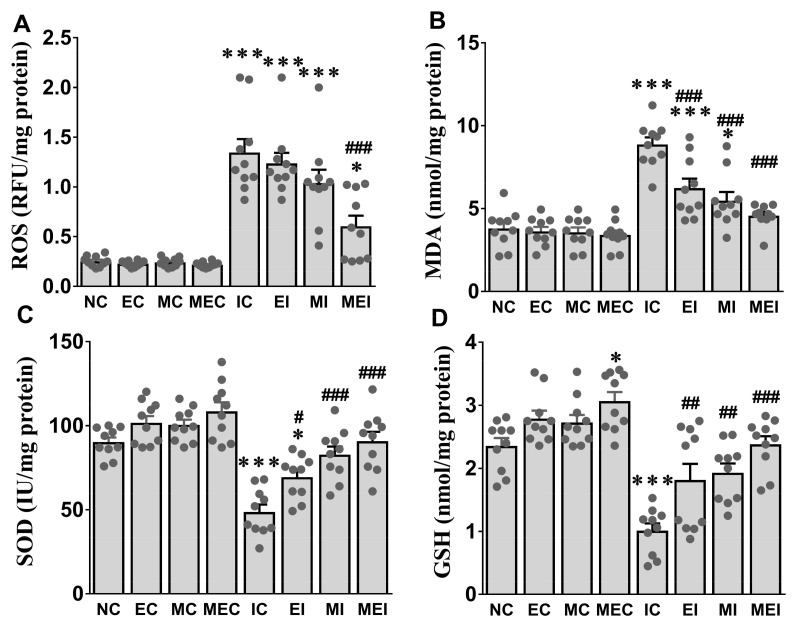
Effect of melatonin and exercise on cardiac oxidative stress-related markers in isoproterenol-challenged rats. (**A**) ROS, reactive oxygen species; (**B**) MDA, malondialdehyde; (**C**) SOD, superoxide dismutase; (**D**) GSH, glutathione. The data are reported as the mean ± SEM (*n* = 10). * *p* < 0.05 and *** *p* < 0.001 versus the NC group. # *p* < 0.05, ## *p* < 0.01, and ### *p* < 0.001 versus the IC group calculated through one-way ANOVA followed by the post hoc Bonferroni test. NC, normal control; EC, exercise control group; MC, melatonin control group; MEC, melatonin plus exercise control group; IC, isoproterenol control group; EI, exercise plus isoproterenol-challenged group; MI, melatonin plus isoproterenol-challenged group; MEI, melatonin and exercise plus isoproterenol-challenged group. ROS, reactive oxygen species; MDA, malondialdehyde; SOD, superoxide dismutase; GSH, glutathione.

The three-week exercise (EC), melatonin-alone (MC), and combination of melatonin and exercise (MEC) groups had the tendency of improved antioxidant activities compared with those in the control group. However, these antioxidant activities were significantly decreased after challenging with isoproterenol (Figure 2). Notably, these alterations were effectively counteracted by melatonin and exercise regimentation in the MEI group (Figure 2).

### 3.4. Effect of Exercise and Melatonin on Mitochondrial Function

**Figure 3:**
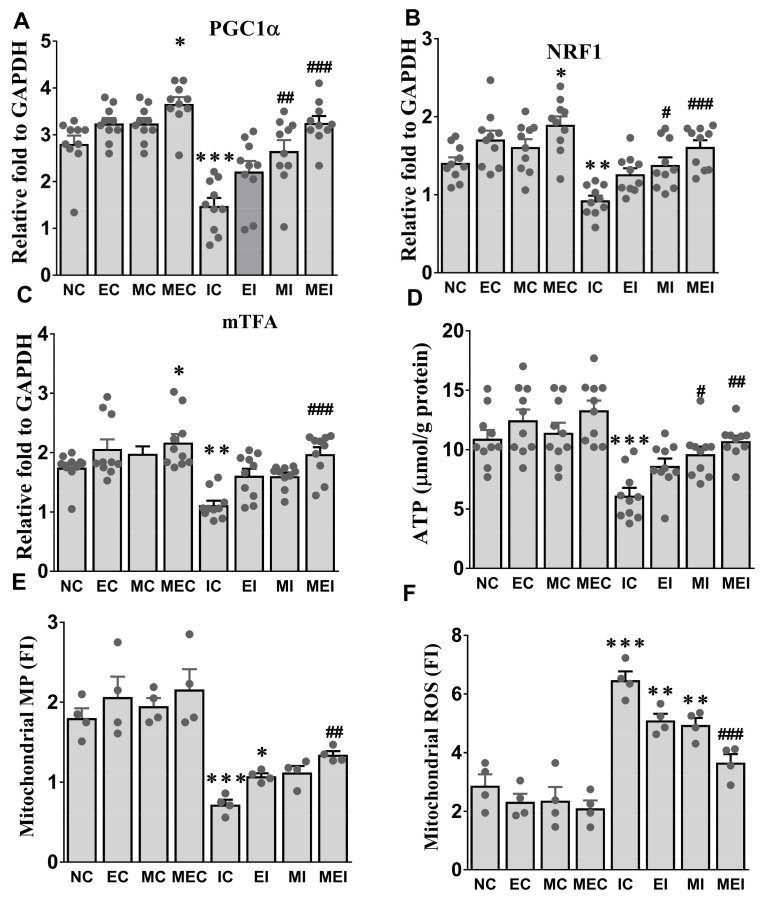
Effect of melatonin and exercise on cardiac mitochondrial function in isoproterenol-challenged rats. (**A**) PGC-1α, Peroxisome proliferator-activated receptor gamma coactivator 1α; (**B**) NRF-1, Nuclear respiratory factor-1; (**C**) mTFA, Mitochondrial transcription factor-A; (**D**) ATP, adenosine triphosphate; (**E**) Mitochondrial MP, membrane potentials; (**F**) Mitochondrial ROS, reactive oxygen species. The data are reported as the mean ± SEM (*n* = 4). * *p* < 0.05, ** *p* < 0.01, and *** *p* < 0.001 versus the NC group. # *p* < 0.05, ## *p* < 0.01, and ### *p* < 0.001 versus the IC group calculated through the Bonferroni post hoc test following one-way ANOVA. ROS, reactive oxygen species; FI, fluorescence intensity; NC, normal control; EC, exercise control group; MC, melatonin control group; MEC, melatonin plus exercise control group; IC, isoproterenol control group; EI, exercise plus isoproterenol-challenged group; MI, melatonin plus isoproterenol-challenged group; MEI, melatonin and exercise plus isoproterenol-challenged group; MMP, mitochondrial membrane potential; ROS, reactive oxygen species; FI, fluorescence intensity.

Melatonin and exercise regimentation markedly up-regulated the expression of PGC-1α along with mitochondrial biogenesis-related genes, such as NRF1, mTFA, tissue ATP, and MMP, in the MEC group and decreased mitochondrial ROS generation. However, isoproterenol exposure significantly reduced the expression of PGC-1α, NRF1, mTFA genes, tissue ATP level, and MMP, and increased mitochondrial ROS, but these alterations were significantly protected against by the combined action of melatonin and exercise in the MEI group (Figure 3).

### 3.5. Effect of Exercise and Melatonin on Inflammatory Cytokine, ER Stress Markers, and Histopathology in Cardiac Tissue

**Figure 4:**
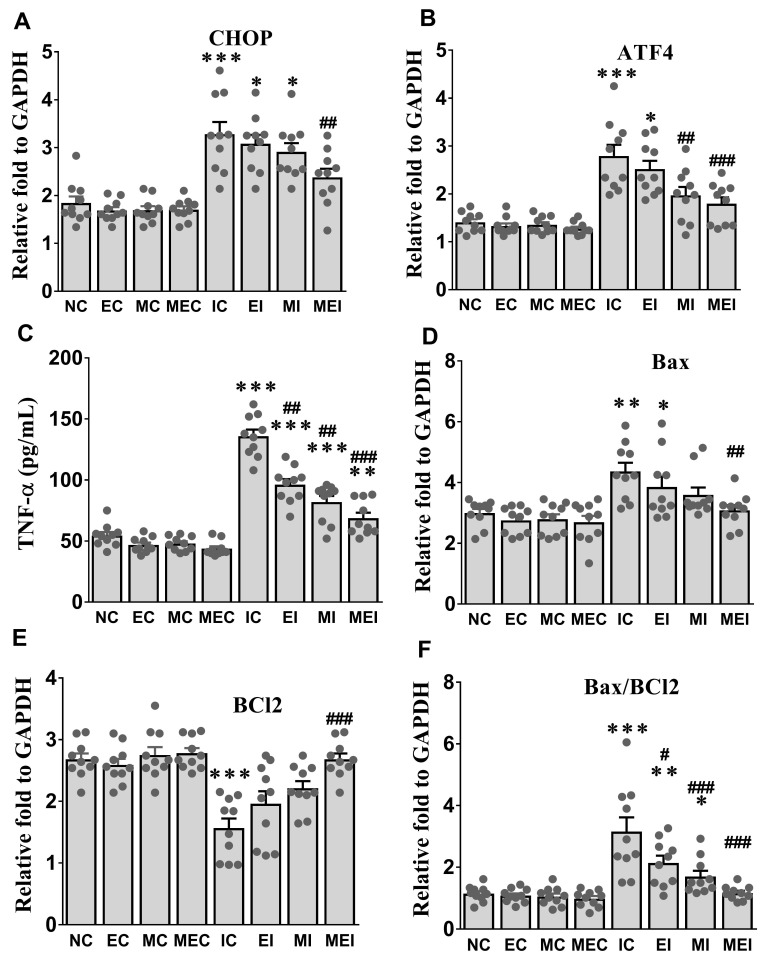
Effect of melatonin and exercise on cardiac ER stress, inflammatory cytokine, and apoptosis-related markers in isoproterenol-challenged rats. (**A**) CHOP, C/EBP homologous protein; (**B**) ATF4, Activating transcription factor 4; (**C**) TNF-α, Tumor necrosis factor alpha; (**D**) Bax, Bcl-2-associated X protein; (**E**) BCL-2, B-cell lymphoma protein 2; (**F**) Bax/BCL-2, the ratio of Bax and BCL-2. The data are reported as the mean ± SEM (*n* = 6). * *p* < 0.05, ** *p* < 0.01, and *** *p* < 0.001 versus the NC group. # *p* < 0.05, ## *p* < 0.01, and ### *p* < 0.001 versus the IC group calculated through the Bonferroni post hoc test following one-way ANOVA. NC, normal control; EC, exercise control group; MC, melatonin control group; MEC, melatonin plus exercise control group; IC, isoproterenol control group; EI, exercise plus isoproterenol-challenged group; MI, melatonin plus isoproterenol-challenged group; MEI, melatonin and exercise plus isoproterenol-challenged group.

**Figure 5:**
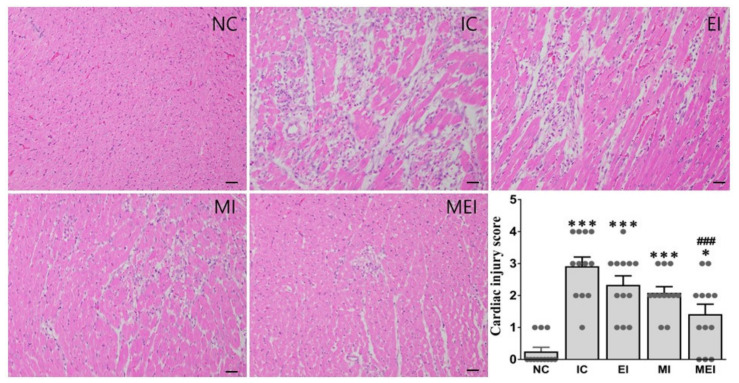
Effect of melatonin and exercise on histopathological changes in heart tissue. H&E staining was performed and visualized under a light microscope at ×200 magnification (*n* = 4 per group). Micrograph shows that tissue necrosis and inflammatory infiltration are markedly elevated in the IC group, whereas these are effectively controlled by combined action of melatonin and exercise, as shown in the MEI group, representing their cardio-protective effect. * *p* < 0.05 and *** *p* < 0.001 versus the NC group. ### *p* < 0.001 versus the IC group calculated through the Bonferroni post hoc test following one-way ANOVA. NC, normal control; IC, isoproterenol control group; EI, exercise plus isoproterenol-challenged group; MI, melatonin plus isoproterenol-challenged group; MEI, melatonin and exercise plus isoproterenol-challenged group. Scale bar, 100 μm.

**Figure 6:**
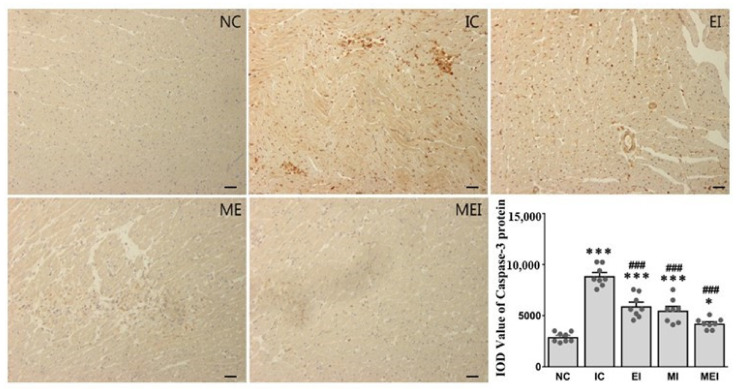
Effect of melatonin and exercise on the expression of caspase-3 protein. Immunohistochemical analysis was performed for staining of caspase-3 protein in the heart (*n* = 4 per group). Micrograph shows that caspase-3-positive cells are markedly increased in the IC group, whereas they are effectively suppressed by combined action of melatonin and exercise in the MEI group, representing their anti-apoptotic effect. * *p* < 0.05 and *** *p* < 0.001 versus the NC group. ### *p* < 0.001 versus the IC group calculated through the Bonferroni post hoc test following one-way ANOVA. NC, normal control; IC, isoproterenol control group; EI, exercise plus isoproterenol-challenged group; MI, melatonin plus isoproterenol-challenged group; MEI, melatonin and exercise plus isoproterenol-challenged group. Scale bar, 100 μm.

**Figure 7:**
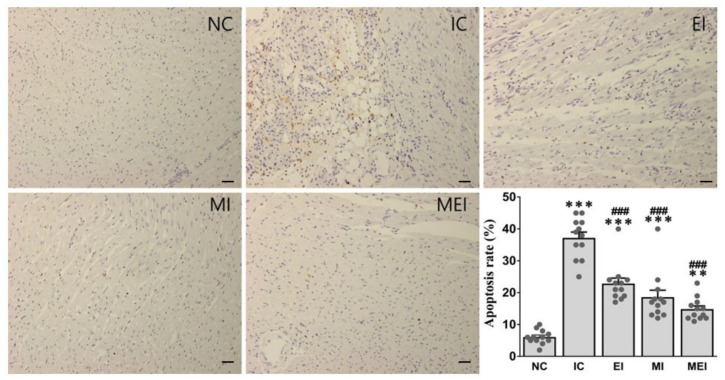
Effects of melatonin and exercise on cardiac apoptotic cell death in isoproterenol-challenged rats. Micrograph shows that TUNEL-positive cells were markedly increased in the IC group, whereas these are effectively inhibited by combined action of melatonin and exercise in the MEI group, indicating their anti-apoptotic effect (*n* = 4 per group). ** *p* < 0.01 and *** *p* < 0.001 versus the NC group. ### *p* < 0.001 versus the IC group calculated through the Bonferroni post hoc test following one-way ANOVA. NC, normal control; IC, isoproterenol control group; EI, exercise plus isoproterenol-challenged group; MI, melatonin plus isoproterenol-challenged group; MEI, melatonin and exercise plus isoproterenol-challenged group. Scale bar, 100 μm.

Alterations of tissue TNF-α, ER stress markers, including CHOP and AFT4, and apoptosis-related genes, including Bax and Bcl2, were also significantly protected against in the MEI group compared with those in the IC group (Figure 4). The protective effects of the combination of melatonin and exercise were further confirmed by histopathological analysis (Figure 5), immunohistochemical analysis of the expression of caspase-3 as an apoptosis-inducible protein (Figure 6), and TUNEL assay for the detection of apoptotic cells (Figure 7).

## 4. Discussion

Melatonin and exercise regimentation improved the antioxidant activities of SOD and GSH, up-regulated PGC-1α and mitochondrial function, and improved ATP contents in the MEC group, which may be act in combination as reserve soldiers to counteract the challenges of isoproterenol-induced tissue injury, oxidative stress, mitochondrial dysfunction, apoptosis, and cardiac dysfunction.

Contractile dysfunction of LV is a major clinical sign of ischemic cardiomyopathies [24,25], which reflected in this study by the altered hemodynamic parameters such as HR, SAP, DAP, MAP, LVESP, LVEDP, and +/− dpdt in the IC group. Additionally, the heart and LV percentages to body and LV percentage to heart also increased, and remarkable histopathological changes were also found in the isoproterenol-challenged IC group. Notably, all these alterations were effectively counteracted by melatonin supplementation and exercise regimentation in the MEI group. Furthermore, lowered body weight was observed in the groups treated with melatonin or exercise alone, indicating that the anti-obesity effects of melatonin and exercise might be manifested in the lowered lipid profiles in these groups compared to the NC group. The relative increase in LV weight in the IC group resulted from increased inflammatory infiltration, edematous swelling, and protein accumulation following isoproterenol-induced injury, while the relative decrease in LV weight in the MEI group might be from controlling the inflammation (TNF-α) and accumulation of cellular infiltration following isoproterenol-induced injury [5]. Severe necrosis was found in the histopathology of the IC group and was effectively protected against in the MEI group. The serum level of CK-MB, cTnI, CK, and CK index are considered as diagnostic markers of myocardial infarction and cardiac tissue injuries [5,25], which all were found to be efficiently lowered in the MEI group and confirmed, again, less cardiac tissue injury.

Unregulated ROS generation can induce oxidative stress, which results in damage to DNA, proteins, and lipids, as well as mitochondrial dysfunction, ATP synthesis, ER stress, and cell death [26,27]. Indeed, oxidative stress plays a critical role in cardiac diseases, such as cardiomyopathy, cardiac ischemia–reperfusion injury, and heart failure [26].

To elucidate the molecular mechanisms involved herein, we investigated the oxidative stress-associated markers, including ROS level, MDA, SOD, and GSH, as well as PGC-1α, mitochondrial biogenesis, ER stress, and apoptosis-related gene expression, in cardiac tissue. The results showed that melatonin and exercise synergistically reduced ROS generation and MDA level in the heart, while antioxidant markers, including SOD and GSH, were significantly higher in the MEI group compared to those in the IC group. Therefore, we concluded that combinatory effects of melatonin and exercise might be responsible for better counteraction against isoproterenol-induced cardiac injury and for better contractile function.

PGC-1α plays a crucial role in energy metabolism modulation and is a prime regulator of mitochondrial biogenesis as well as a regulator of cardiac oxidative phosphorylation and reactive oxygen species detoxification [8]. Furthermore, previous studies reported that the expression of this protein is altered in human cardiac diseases [21] and isoproterenol-induced cardiomyopathy models in rats [4]. Consistently, we also found that the expression of PGC-1α was down-regulated, accompanied with the down-regulation of mitochondrial biogenesis-related genes, such as NRF1 and mTFA, in the IC group compared to those in the NC group. Melatonin [13,28,29] and exercise [30,31] are well known to up-regulate PGC-1α as well as increase mitochondrial biogenesis in various tissues. Interestingly, our results showed that the combination of melatonin and exercise synergistically up-regulated PGC-1α and mitochondrial biogenesis in the MEC group, and these were found to be significantly higher in the MEI group, even after challenging with isoproterenol, than those in the IC group. Therefore, the up-regulation of PGC-1α in the MEI group may improve the antioxidant activities [32], as it acts as an antioxidant enhancer [13]. Consequently, the intracellular ROS and MDA levels were decreased.

Indeed, the contractile function of the beating heart is governed by the constant flux of ATP, and it is logical that a lack of ATP or energetic defects contributes to the advancement of contractile dysfunction and heart failure [33,34]. Mitochondria are vital organelles that play essential roles in ATP production, energy transduction, and numerous cellular signaling phenomena. Mitochondrial biogenesis is critical to orchestrate mitochondria, which are important to continuously supply ATP to cardiac tissue for maintaining normal function [33,34]. Mitochondrial ROS production, mitochondrial membrane potential, and cellular ATP changes were evaluated to assess the mitochondrial function of the myocardium. In this study, preserved mitochondrial functions were shown, evidenced by the up-regulation of mitochondrial biogenesis-related genes, including NRF1, mTFA, and PGC-1α, but not significantly in the MEC group, along with higher ATP content in cardiac tissue compared to those in the sedentary NC, EC, or MC group. Importantly, the ATP level, PGC-1α, NRF1, and mTFA gene expression were lowered in the IC group, and mostly ameliorated in the MEI group. Moreover, the mitochondrial membrane potential was significantly higher and ROS level was significantly lower in the MEI group, indicating the underlying mechanism of melatonin and exercise for the suppression of oxidative stress and injury and better contractile function of the heart. Because the dysfunction of cardiac mitochondria leads to reduced ATP synthesis, the excessive formation of ROS and lipid peroxidation thus imbalanced the antioxidants, in turn, inducing contractile dysfunction and heart failure [34,35].

Apoptosis plays a vital role for cardiac dysfunction [36]. It can occur following ischemia or infarction, which might be due to experiencing moderate oxidative and ER stress through various finely tuned molecular signals [37,38]. Although a large number of genes are involved in regulating apoptotic cell death, the antiapoptotic Bcl-2 and proapoptotic Bax proteins play major roles in the induction of myocardial apoptosis following ischemia and reperfusion. Moreover, apoptotic cell death is terminated by a family of aspartate-specific cysteine proteases known as caspases. Among them, caspase-3 plays a crucial role in cardiomyocyte apoptosis, which denotes the final pathway of the caspase cascade [33]. In this study, we observed that increased oxidative stress, which may induce cell apoptosis in cardiac tissue, is reflected by the decrease in the Bcl-2/Bax expression ratio and increase in caspase-3 protein expression in the IC group, which were protected against by the joint action of melatonin and exercise in the MEI group.

ER stress plays a critical role in cardiomyocytes to induce apoptosis and inflammatory and metabolic pathologies, which contribute to cardiac dysfunction [6,39,40]. The ER stress markers, including ATF6 and CHOP, along with caspase-3 were elevated in cardiac tissue after challenging with isoproterenol in this study. It has been reported that CHOP expression is the most sensitive tool for ER stress, activating caspase-3 to amplify the apoptosis cascade through various signals [6,40]. Elevated levels of the cardiac inflammatory cytokine TNF-α were observed after isoproterenol administration in this study. It has been reported that TNF-α can trigger apoptosis via the mitochondrial signaling pathway [38]. Moreover, the overexpression of inflammatory cytokines is responsible for progressive cardiac decompensation and clinical features of heart failure [5]. Notably, melatonin and exercise reduced ER stress and inflammation along with up-regulation of antioxidant activities, PGC-1α, and mitochondrial biogenesis, thereby preventing cellular apoptosis and inflammation. Because of experiencing the extreme oxidative stress, the mitochondrial permeability transition increased, causing the interruption of the intracellular supply of ATP, which, in turn, leads to necrotic cell death [37].

## 5. Conclusions

**Figure 8:**
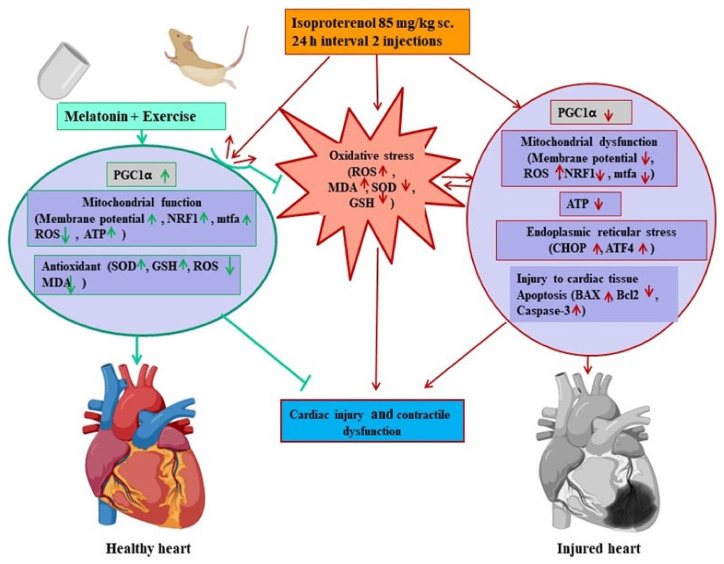
Schematic diagram of the proposed mechanisms by which melatonin and exercise counteract the challenges of isoproterenol-induced cardiac oxidative stress, injury, and contractile dysfunction. In rats, melatonin supplementation and exercise improved anti-oxidative activities, up-regulated PGC-1α and mitochondrial biogenesis, and improved ATP, which may act in combination as reserve soldiers to counteract the challenges of isoproterenol-induced oxidative stress, down-regulation of PGC-1α, mitochondrial function, ATP interruption, ER stress, and apoptosis, thereby preventing cardiac injury and contractile dysfunction.

Finally, this study demonstrated that cardiac oxidative stress, mitochondrial dysfunction, ATP interruption, inflammation, ER stress, and apoptosis act in combination to induce cardiac dysfunction and injury after isoproterenol administration in rats. All of these alterations were effectively counteracted by a combination of melatonin supplementation and exercise, evidenced by the suppression of ROS generation, MDA production, improved enzymatic antioxidant SOD and non-enzymatic antioxidant GSH level, improved mitochondrial function, and ATP production, which were responsible for counteracting tissue injury and proper contractile function in the MEI group (Figure 8). Therefore, regular exercise and melatonin supplementation could be a promising method to protect against sudden cardiac injury and failure.

## Figures and Tables

**Table 1:** Primers used for real-time PCR amplification.

Genes	Primers	
*PGC1-α*	forward	5′-ACCCACAGGATCAGAACAAACC-3′
	reverse	5′-GACAAATGCTCTTTGCTTTATTGC-3′
*NRF-1*	forward	5′-TTCTCTGCTGTGGCTGATGG-3′
	reverse	5′-CCTCTGATGCTTGCGTCGTCT-3′
*mtTFA*	forward	5′-AGCCATGTGGAGGGAGCTT-3′
	reverse	5′-TTGTACACCTTCCACTCAGCTTTAA-3′
*CHOP*	forward	5′-CCAGCAGAGGTCACAAGCAC-3′
	reverse	5′-CGCACTGACCACTCTGTTTC-3′
*ATF4*	forward	5′-GTTGGTCAGTGCCTCAGACA-3′
	reverse	5′-CATTCGAAACAGAGCATCGA-3′
*Bcl2*	forward	5′-ATGTGTGTGGAGAGCGTCAACC-3′
	reverse	5′-CCAGGAGAAATCAAACAGAGGC-3′
*Bax*	forward	5′-GCGATGAACTGGACAACAACAT-3′
	reverse	5′-TAGCAAAGTAGAAAAGGGCAACC-3′
*GAPDH*	forward	5′-TTCTTGTGCAGTGCCAGCCTCGTC-3′
	reverse	5′-TAGGAACACGGAAGGCCATGCCAG-3′

**Table 2:** Effect of exercise and melatonin supplementation on body and heart tissue weight in rats with or without isoproterenol challenge.

	BW(g)	HW (g)	HW to BW (%)	LVW (g)	LVW to BW (%)	LVW to HW (%)
NC	372 ± 3	1.22 ± 0.04	0.33 ± 0.01	0.85 ± 0.04	0.23 ± 0.01	69 ± 1
EC	351 ± 3 *#	1.21 ± 0.04	0.34 ± 0.01	0.84 ± 0.04	0.24 ± 0.01	69 ± 1
MC	357 ± 6	1.17 ± 0.02	0.33 ± 0.01	0.80 ± 0.02	0.23 ± 0.01	68 ± 1
MEC	349 ± 3 *#	1.21 ± 0.02	0.35 ± 0.01	0.84 ± 0.02	0.24 ± 0.01	69 ± 1
IC	363 ± 2	1.40 ± 0.02 **	0.38 ± 0.00 ***	1.03 ± 0.02 ***	0.28 ± 0.01 ***	73 ± 1 **
EI	349 ± 5 *#	1.28 ± 0.04 #	0.37 ± 0.01 #	0.91 ± 0.04 #	0.26 ± 0.01	71 ± 1 #
MI	354 ± 4	1.27 ± 0.02 #	0.36 ± 0.01 ##	0.90 ± 0.02 #	0.25 ± 0.01 #	71 ± 1 #
MEI	350 ± 2 *#	1.22 ± 0.02 ##	0.35 ± 0.01 ###	0.85 ± 0.02 ###	0.24 ± 0.01 ##	70 ± 1 ##

The data are reported as the mean ± SEM (n = 10). * *p* < 0.05, ** *p* < 0.01, and *** *p* < 0.001 versus the NC group. # *p* < 0.05, ## *p* < 0.01, and ### *p* < 0.001 versus the IC group calculated through one-way ANOVA followed by the post hoc Bonferroni test. NC, normal control; EC, exercise control group; MC, melatonin control group; MEC, melatonin plus exercise control group; IC, isoproterenol control group; EI, exercise plus isoproterenol-challenged group; MI, melatonin plus isoproterenol-challenged group; MEI, melatonin and exercise plus isoproterenol-challenged group. BW, body weight; HW, heart weight; LVW, left ventricular weight.

**Table 3:** Effect of exercise and melatonin supplementation on serum biochemistry in rats with or without isoproterenol challenge.

	NC	EC	MC	MEC	IC	EI	MI	MEI
Troponin I (pg/mL)	151 ± 18	116 ± 13	121 ± 13	113 ± 18	510 ± 57 ***	375 ± 61 **	319 ± 24 *	218 ± 23
CK-MB (ng/mL)	7.53 ± 0.56	6.13 ± 0.36	6.63 ± 0.36	5.53 ± 0.54	16.02 ± 1.10 ***	11.56 ± 0.41 ***#	9.77 ± 0.24 **##	9.08 ± 0.44 *###
CK (IU/mL)	272 ± 16	270 ± 13	265 ± 19	261 ± 15	507 ± 50 ***	443 ± 44 **	353 ± 28 **##	338 ± 15 ##
CK-Index	2.8 ± 0.2	2.3 ± 0.2	2.5 ± 0.3	2.1 ± 0.2 *	3.5 ± 0.4 *	2.8 ± 0.3	2.9 ± 0.3	2.7 ± 0.2
LDH (IU/mL)	202 ± 12	198 ± 5	199 ± 14	195 ± 14	381 ± 14 ***	352 ± 16 ***	224 ± 11 ###	213 ± 12 ###
AST (IU/mL)	130 ± 4	128 ± 6	125 ± 6	124 ± 6	304 ± 22 ***	253 ± 13 ***	170 ± 8 ***###	146 ± 7 ###
TC (mg/dL)	159 ± 3	144 ± 5 *	150 ± 9	143 ± 8 *	166 ± 6 ***	155 ± 5 ***	150 ± 5	149 ± 4
HDL (mg/dL)	36 ± 3	42 ± 3	35 ± 2	42 ± 3	35 ± 2	39 ± 2	35 ± 2	41 ± 3
LDL (mg/dL)	16 ± 1	12 ± 1 *	12 ± 1 *	10 ± 1 *	17 ± 2 *	13 ± 1 *#	13 ± 1 *#	11 ± 1 *##
TG (mg/dL)	142 ± 4	132 ± 3	127 ± 2 *	117 ± 6 **	147 ± 3	140 ± 5	128 ± 4 *#	119 ± 3 **##
AI (mg/dL)	4.63 ± 0.40	3.56 ± 0.26 *	4.40 ± 0.33	3.54 ± 0.24 *	4.91 ± 0.47 *	4.02 ± 0.48 ***	4.53 ± 0.27	3.80 ± 0.28 #

The data are reported as the mean ± SEM (n = 10). * *p* < 0.05, ** *p* < 0.01, and *** *p* < 0.001 versus the NC group. # *p* < 0.05, ## *p* < 0.01, and ### *p* < 0.001 versus the IC group calculated through one-way ANOVA followed by the post hoc Bonferroni test. NC, normal control; EC, exercise control group; MC, melatonin control group; MEC, melatonin plus exercise control group; IC, isoproterenol control group; EI, exercise plus isoproterenol-challenged group; MI, melatonin plus isoproterenol-challenged group; MEI, melatonin and exercise plus isoproterenol-challenged group. CK, creatine kinase; LDH, lactate dehydrogenase; AST, aspartate aminotransferase; TC, total cholesterol; HDL, high-density lipoprotein; LDL, low-density lipoprotein; TG, triglyceride; AI, atherogenic index.

## Data Availability

Data are contained within the article.

## References

[1] Ghorpade A.G., Shrivastava S.R., Kar S.S., Sarkar S., Majgi S.M., Roy G. (2015). Estimation of the cardiovascular risk using World Health Organization/International Society of Hypertension (WHO/ISH) risk prediction charts in a rural population of South India. Int. J. Health Policy Manag..

[2] Rahman M.M., Kwon H.S., Kim M.J., Go H.K., Oak M.H., Kim D.H. (2017). Melatonin supplementation plus exercise behavior ameliorate insulin resistance, hypertension and fatigue in a rat model of type 2 diabetes mellitus. Biomed. Pharmacother. Biomed. Pharmacother..

[3] Mendis S., Thygesen K., Kuulasmaa K., Giampaoli S., Mahonen M., Ngu Blackett K., Lisheng L., Writing group on behalf of the participating experts of the WHO consultation for revision of WHO definition of myocardial infarction (2011). World Health Organization definition of myocardial infarction: 2008–09 revision. Int. J. Epidemiol..

[4] Zhang S., Tang F., Yang Y., Lu M., Luan A., Zhang J., Yang J., Wang H. (2015). Astragaloside IV protects against isoproterenol-induced cardiac hypertrophy by regulating NF-kappaB/PGC-1alpha signaling mediated energy biosynthesis. PLoS ONE.

[5] Patel V., Upaganlawar A., Zalawadia R., Balaraman R. (2010). Cardioprotective effect of melatonin against isoproterenol induced myocardial infarction in rats: A biochemical, electrocardiographic and histoarchitectural evaluation. Eur. J. Pharmacol..

[6] Zhuo X.Z., Wu Y., Ni Y.J., Liu J.H., Gong M., Wang X.H., Wei F., Wang T.Z., Yuan Z., Ma A.Q. (2013). Isoproterenol instigates cardiomyocyte apoptosis and heart failure via AMPK inactivation-mediated endoplasmic reticulum stress. Apoptosis Int. J. Program. Cell Death.

[7] Munzel T., Camici G.G., Maack C., Bonetti N.R., Fuster V., Kovacic J.C. (2017). Impact of Oxidative Stress on the Heart and Vasculature: Part 2 of a 3-Part Series. J. Am. Coll. Cardiol..

[8] Rius-Perez S., Torres-Cuevas I., Millan I., Ortega A.L., Perez S. (2020). PGC-1alpha, Inflammation, and Oxidative Stress: An Integrative View in Metabolism. Oxid. Med. Cell Longev..

[9] Buttar H.S., Li T., Ravi N. (2005). Prevention of cardiovascular diseases: Role of exercise, dietary interventions, obesity and smoking cessation. Exp. Clin. Cardiol..

[10] Adhihetty P.J., O’Leary M.F., Chabi B., Wicks K.L., Hood D.A. (2007). Effect of denervation on mitochondrially mediated apoptosis in skeletal muscle. J. Appl. Physiol..

[11] Rahman M.M., Lee S.J., Mun A.R., Adam G.O., Park R.M., Kim G.B., Kang H.S., Kim J.S., Kim S.J., Kim S.Z. (2014). Relationships between blood Mg2+ and energy metabolites/enzymes after acute exhaustive swimming exercise in rats. Biol. Trace Elem. Res..

[12] Garcia-Mesa Y., Gimenez-Llort L., Lopez L.C., Venegas C., Cristofol R., Escames G., Acuna-Castroviejo D., Sanfeliu C. (2012). Melatonin plus physical exercise are highly neuroprotective in the 3xTg-AD mouse. Neurobiol. Aging.

[13] Carloni S., Albertini M.C., Galluzzi L., Buonocore G., Proietti F., Balduini W. (2014). Melatonin reduces endoplasmic reticulum stress and preserves sirtuin 1 expression in neuronal cells of newborn rats after hypoxia-ischemia. J. Pineal Res..

[14] Raygan F., Ostadmohammadi V., Bahmani F., Reiter R.J., Asemi Z. (2019). Melatonin administration lowers biomarkers of oxidative stress and cardio-metabolic risk in type 2 diabetic patients with coronary heart disease: A randomized, double-blind, placebo-controlled trial. Clin. Nutr..

[15] El Assar M., Alvarez-Bustos A., Sosa P., Angulo J., Rodriguez-Manas L. (2022). Effect of Physical Activity/Exercise on Oxidative Stress and Inflammation in Muscle and Vascular Aging. Int. J. Mol. Sci..

[16] Yeleswaram K., McLaughlin L.G., Knipe J.O., Schabdach D. (1997). Pharmacokinetics and oral bioavailability of exogenous melatonin in preclinical animal models and clinical implications. J. Pineal. Res..

[17] Rahman M.M., Park S.J., Jeon H.Y., Kim S. (2021). Exercise and oral melatonin attenuate anxiety and depression like behavior in type 2 diabetic rats. J. Adv. Biotechnol. Exp. Ther..

[18] Fan Y. (2019). Cardioprotective Effect of Rhapontigenin in Isoproterenol-Induced Myocardial Infarction in a Rat Model. Pharmacology.

[19] Chen Y., Gui D., Chen J., He D., Luo Y., Wang N. (2014). Down-regulation of PERK-ATF4-CHOP pathway by Astragaloside IV is associated with the inhibition of endoplasmic reticulum stress-induced podocyte apoptosis in diabetic rats. Int. J. Exp. Cell. Physiol. Biochem. Pharmacol..

[20] Wang Y., Liu X., Zhang D., Chen J., Liu S., Berk M. (2013). The effects of apoptosis vulnerability markers on the myocardium in depression after myocardial infarction. BMC Med..

[21] Guo R., Liu B., Zhou S., Zhang B., Xu Y. (2013). The protective effect of fasudil on the structure and function of cardiac mitochondria from rats with type 2 diabetes induced by streptozotocin with a high-fat diet is mediated by the attenuation of oxidative stress. Biomed. Res. Int..

[22] Lu C., Chen C., Chen A., Wu Y., Wen J., Huang F., Zeng Z. (2020). Oridonin Attenuates Myocardial Ischemia/Reperfusion Injury via Downregulating Oxidative Stress and NLRP3 Inflammasome Pathway in Mice. Evid.-Based Complement. Alternat. Med..

[23] Li H., Xie Y.H., Yang Q., Wang S.W., Zhang B.L., Wang J.B., Cao W., Bi L.L., Sun J.Y., Miao S. (2012). Cardioprotective effect of paeonol and danshensu combination on isoproterenol-induced myocardial injury in rats. PLoS ONE.

[24] Pisano A., Cerbelli B., Perli E., Pelullo M., Bargelli V., Preziuso C., Mancini M., He L., Bates M.G., Lucena J.R. (2016). Impaired mitochondrial biogenesis is a common feature to myocardial hypertrophy and end-stage ischemic heart failure. Cardiovasc. Pathol. Off. J. Soc. Cardiovasc. Pathol..

[25] Sachdeva J., Tanwar V., Golechha M., Siddiqui K.M., Nag T.C., Ray R., Kumari S., Arya D.S. (2012). *Crocus sativus* L. (saffron) attenuates isoproterenol-induced myocardial injury via preserving cardiac functions and strengthening antioxidant defense system. Exp. Toxicol. Pathol. Off. J. Ges. Toxikol. Pathol..

[26] Peoples J.N., Saraf A., Ghazal N., Pham T.T., Kwong J.Q. (2019). Mitochondrial dysfunction and oxidative stress in heart disease. Exp. Mol. Med..

[27] Souza-Neto F.V., Islas F., Jimenez-Gonzalez S., Luaces M., Ramchandani B., Romero-Miranda A., Delgado-Valero B., Roldan-Molina E., Saiz-Pardo M., Ceron-Nieto M.A. (2022). Mitochondrial Oxidative Stress Promotes Cardiac Remodeling in Myocardial Infarction through the Activation of Endoplasmic Reticulum Stress. Antioxidants.

[28] Kang J.W., Hong J.M., Lee S.M. (2016). Melatonin enhances mitophagy and mitochondrial biogenesis in rats with carbon tetrachloride-induced liver fibrosis. J. Pineal Res..

[29] Yu L., Gong B., Duan W., Fan C., Zhang J., Li Z., Xue X., Xu Y., Meng D., Li B. (2017). Melatonin ameliorates myocardial ischemia/reperfusion injury in type 1 diabetic rats by preserving mitochondrial function: Role of AMPK-PGC-1alpha-SIRT3 signaling. Sci. Rep..

[30] Arany Z., He H., Lin J., Hoyer K., Handschin C., Toka O., Ahmad F., Matsui T., Chin S., Wu P.H. (2005). Transcriptional coactivator PGC-1 alpha controls the energy state and contractile function of cardiac muscle. Cell Metab..

[31] Lira V.A., Benton C.R., Yan Z., Bonen A. (2010). PGC-1alpha regulation by exercise training and its influences on muscle function and insulin sensitivity. Am. J. Physiol. Endocrinol. Metab..

[32] Baldelli S., Aquilano K., Ciriolo M.R. (2014). PGC-1alpha buffers ROS-mediated removal of mitochondria during myogenesis. Cell Death Dis..

[33] Rowe G.C., Jiang A., Arany Z. (2010). PGC-1 coactivators in cardiac development and disease. Circ. Res..

[34] Brown D.A., Perry J.B., Allen M.E., Sabbah H.N., Stauffer B.L., Shaikh S.R., Cleland J.G., Colucci W.S., Butler J., Voors A.A. (2017). Expert consensus document: Mitochondrial function as a therapeutic target in heart failure. Nat. Rev. Cardiol..

[35] Fabregat-Andres O., Tierrez A., Mata M., Estornell-Erill J., Ridocci-Soriano F., Monsalve M. (2011). Induction of PGC-1alpha expression can be detected in blood samples of patients with ST-segment elevation acute myocardial infarction. PLoS ONE.

[36] Fu H.Y., Okada K., Liao Y., Tsukamoto O., Isomura T., Asai M., Sawada T., Okuda K., Asano Y., Sanada S. (2010). Ablation of C/EBP homologous protein attenuates endoplasmic reticulum-mediated apoptosis and cardiac dysfunction induced by pressure overload. Circulation.

[37] Nishida K., Yamaguchi O., Otsu K. (2008). Crosstalk between autophagy and apoptosis in heart disease. Circ. Res..

[38] Nikoletopoulou V., Markaki M., Palikaras K., Tavernarakis N. (2013). Crosstalk between apoptosis, necrosis and autophagy. Biochim. Biophys. Acta.

[39] Abbate A., Bussani R., Amin M.S., Vetrovec G.W., Baldi A. (2006). Acute myocardial infarction and heart failure: Role of apoptosis. Int. J. Biochem. Cell Biol..

[40] Wu J., Ruas J.L., Estall J.L., Rasbach K.A., Choi J.H., Ye L., Bostrom P., Tyra H.M., Crawford R.W., Campbell K.P. (2011). The unfolded protein response mediates adaptation to exercise in skeletal muscle through a PGC-1alpha/ATF6alpha complex. Cell Metab..

